# Superior Medial-Based Breast Reduction with Inverted T Incision: A Precise Description of Our Refined Preoperative Marking and Operative Technique

**DOI:** 10.1007/s00266-024-04055-5

**Published:** 2024-05-03

**Authors:** Monika Lanthaler, Alexander Haim, Theresia Steinkellner, Carina Harasser, Christoph Tasch, Agnese Nitto, Anton Schwabegger, Dolores Wolfram

**Affiliations:** Department of Plastic, Reconstructive and Aesthetic Surgery, Innsbruck Medical University Hospital, Anichstrasse 35, 6020 Innsbruck, Austria

**Keywords:** Technical refinements, Marking, Operation, Breast reduction

## Abstract

**Abstract:**

The purpose of this paper was to compile a thoroughly elaborated step-by-step guide for the preoperative marking and operative technique for superior medial pedicle inverted T breast reduction based on our long experience and technical refinements.

**Level of Evidence IV:**

This journal requires that authors assign a level of evidence to each article. For a full description of these Evidence-Based Medicine ratings, please refer to the Table of Contents or the online Instructions to Authors www.springer.com/00266.

## Background

Severe breast hypertrophy leads to neck, shoulder and back pain and is often associated with decreased self-esteem. Reduction mammoplasty has been shown to improve physical symptoms as well as self-esteem [1–3]. Although breast reduction looks like cosmetic surgery, it is in fact reconstructive surgery that aims to eliminate the above-mentioned physical disorders [4] so that with eased breathing and more physical mobility without the heavy breast patients can lead a healthier, more fulfilling life.

Of the various breast reduction techniques, the superior medial pedicle method described here is increasingly mentioned in the literature as providing superior esthetic results and better long-term durability. Moreover, shorter operative time is also reported [5–7].

The aim of this paper is to provide a thoroughly elaborated step-by-step guide for the preoperative marking and operative technique for the superior medial inverted T breast reduction refined by us over long years of experience. After reading this paper, it should be simple for every surgeon to follow the described steps with all the mentioned refinements and to also teach the surgical intervention in a clear and straightforward manner to residents.

## Preoperative Marking

**Fig. 1:**
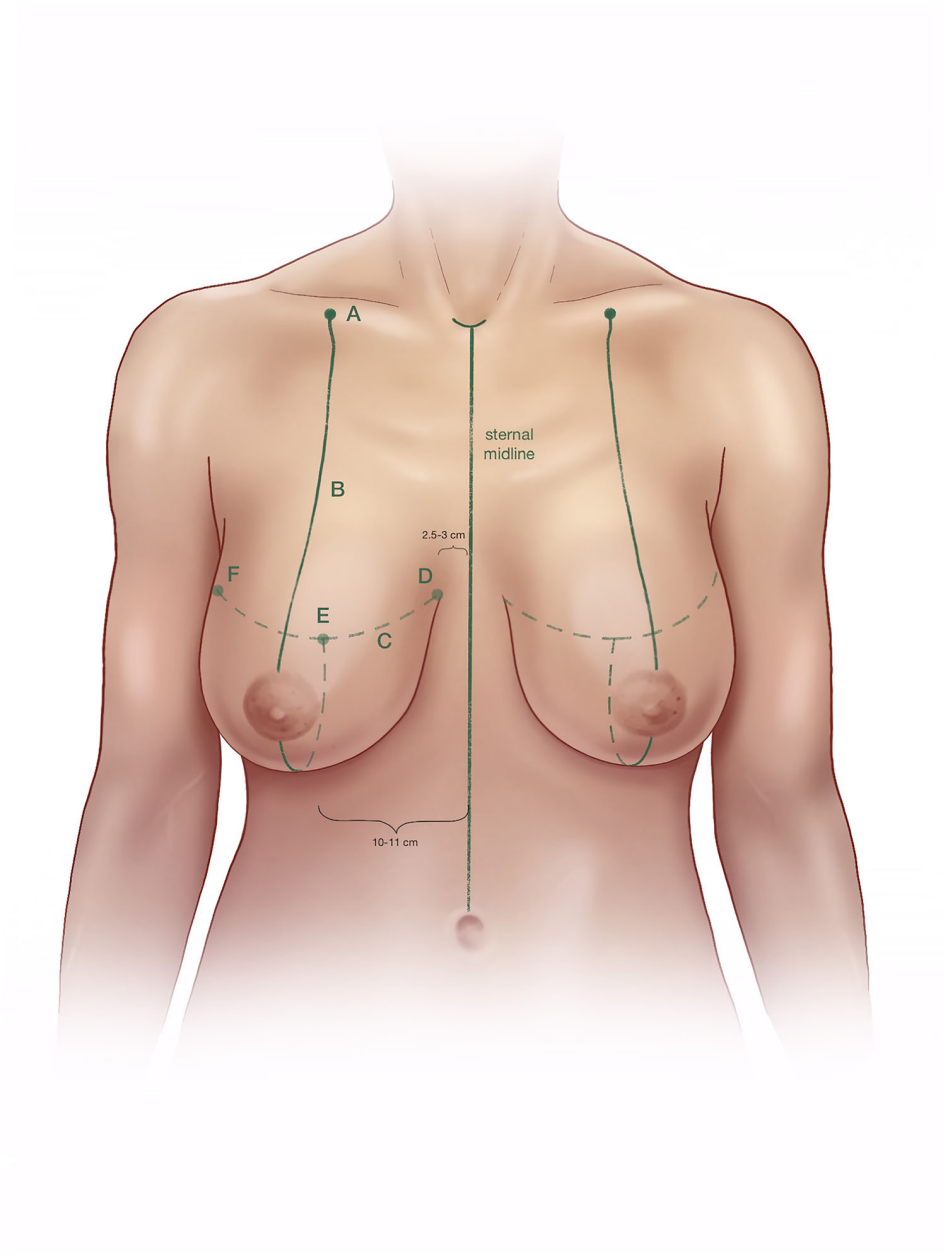
Preoperative markings—important lines

**Fig. 2:**
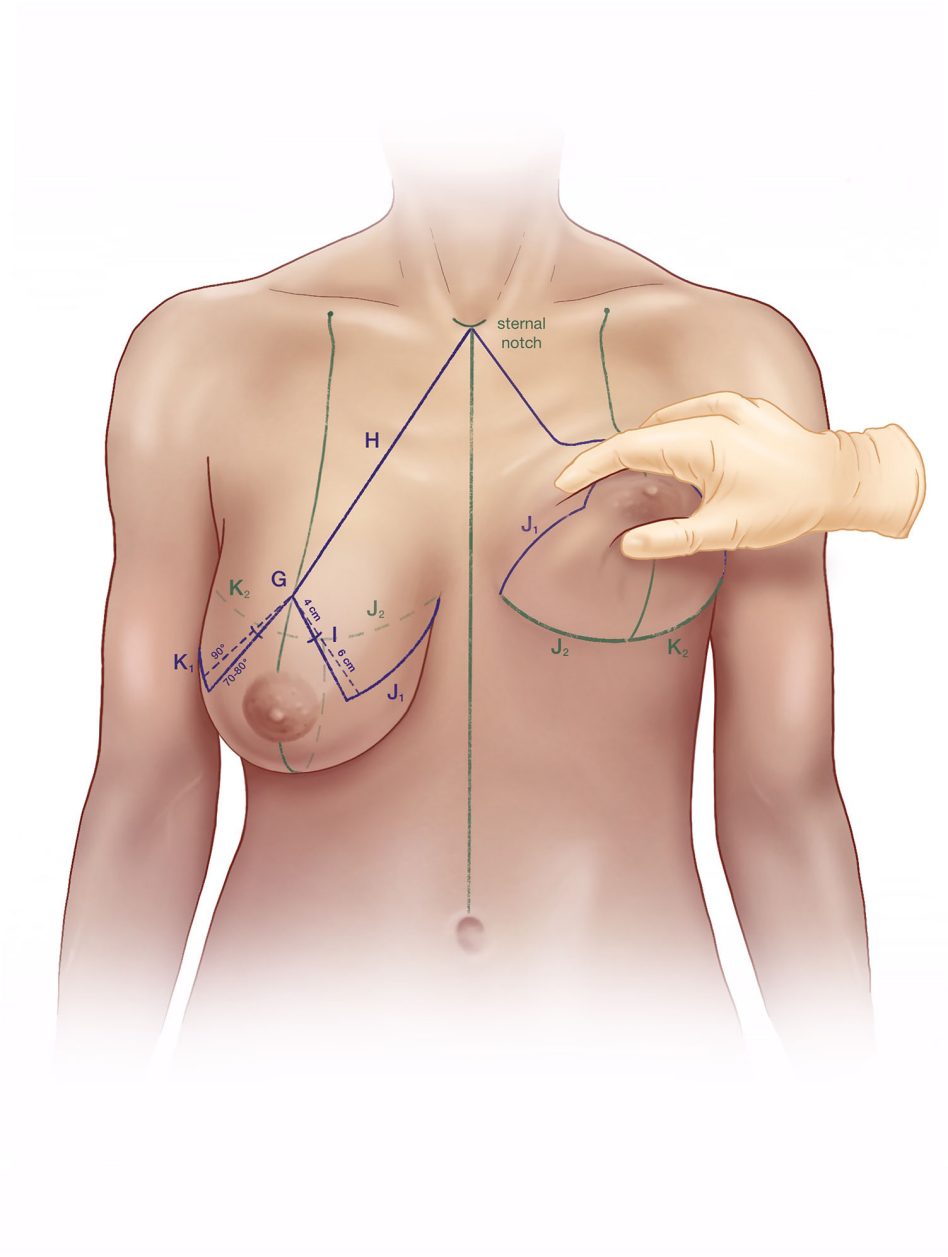
Preoperative markings—vertical sides, horizontal lines

**Fig. 3:**
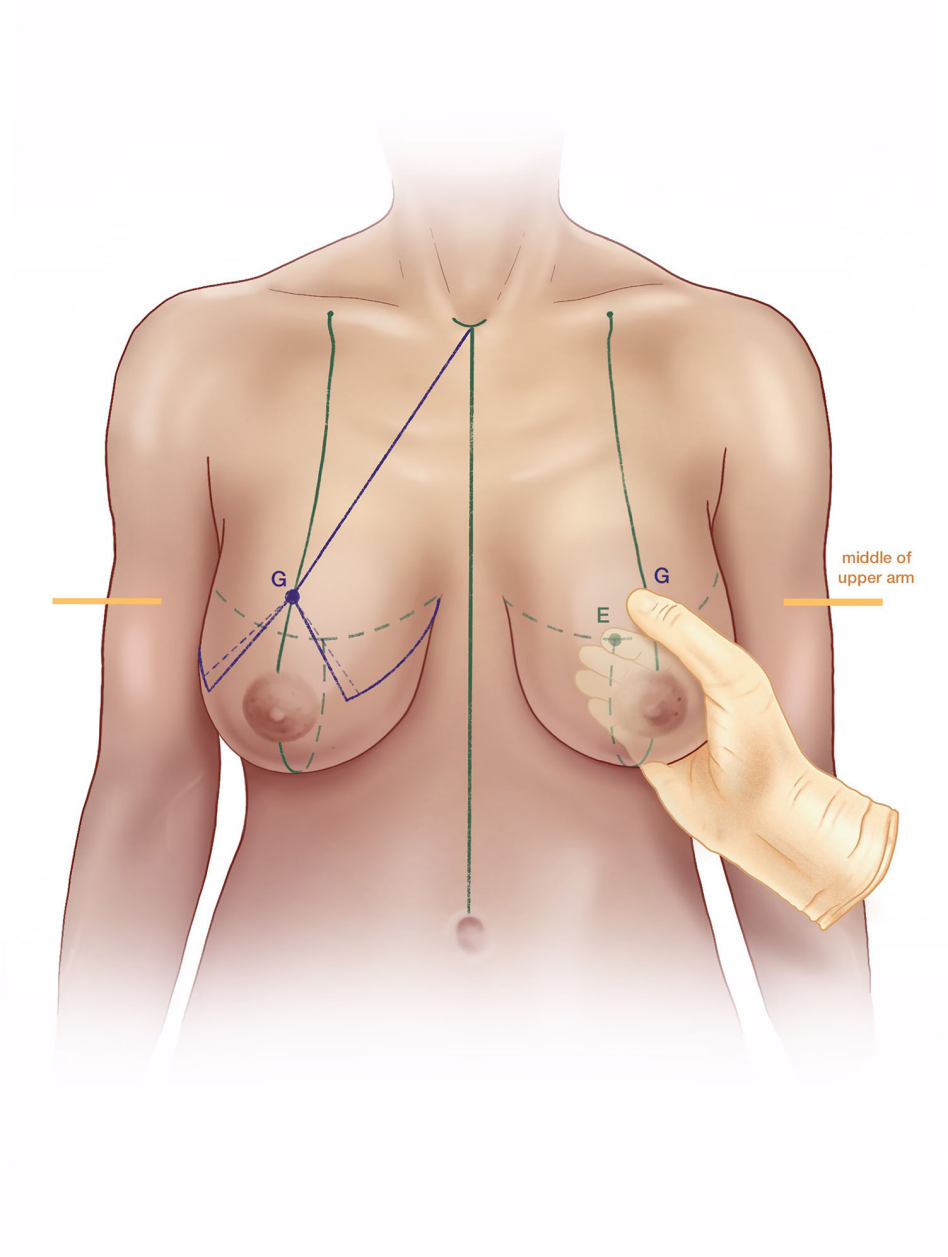
Preoperative markings—upper border of the areola opening, pinch test, middle of the upper arm

**Fig. 4:**
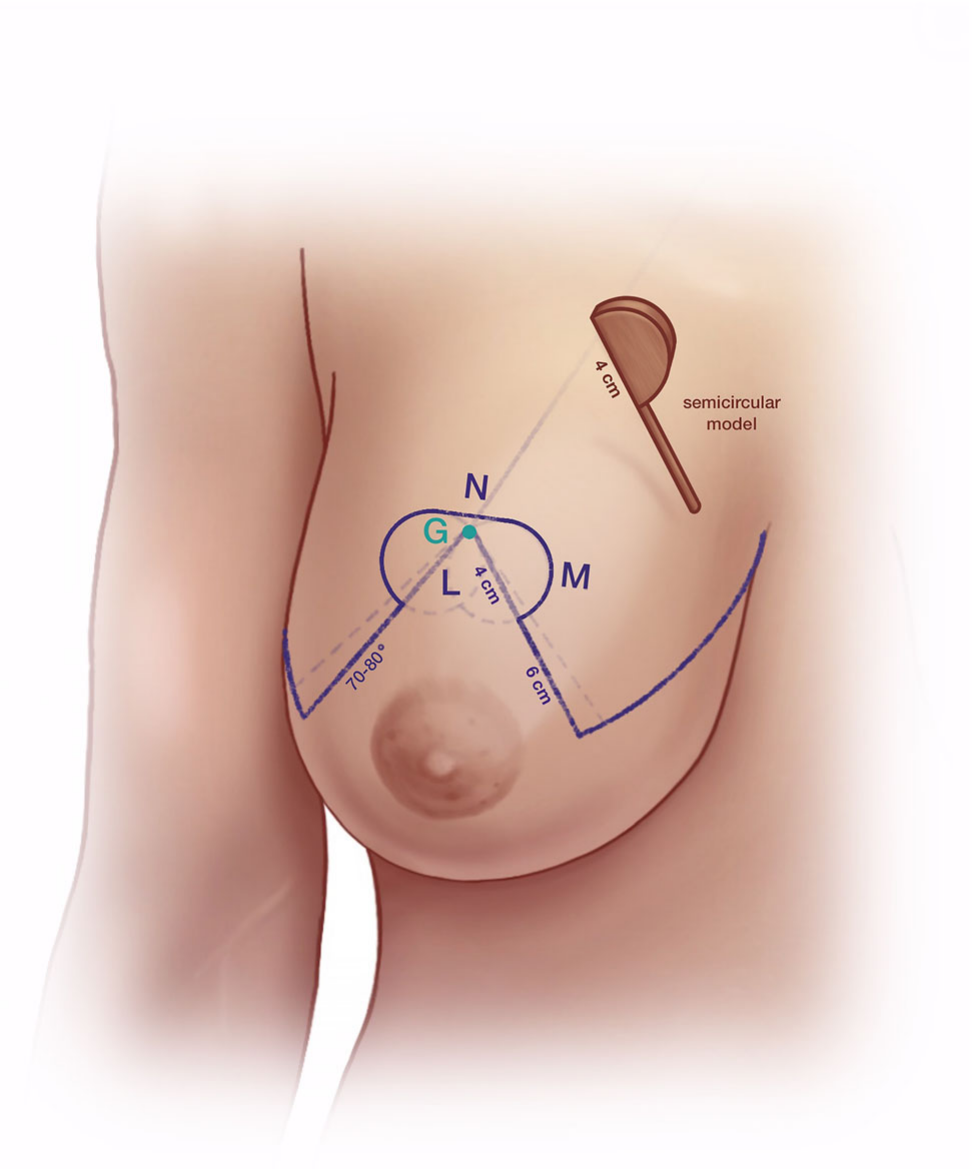
Preoperative markings—areola opening, angle between the vertical sides, 4 cm semicircular template

(A) Mark a point about 7 cm lateral to the sternal notch along the clavicle (Fig. 1).
(B) Draw a line from Point A through the nipple downward through the breast meridian.
(C) Lift the breast and mark the inframammary fold (IMF).
(D) Mark Point D about 2.5–3 cm lateral to the midline so that it crosses the IMF medially with the aim of shifting the most medial end of the inframammary scar away from the sternal midline.
(E) Line B crosses the IMF through the breast meridian about 10–11 cm from the sternal midline, in thin patients sometimes at a shorter distance.
(F) Laterally the IMF should go slightly upward following the natural breast shape. If the natural breast shape does not go slightly upward, especially in very large breasts, draw the IMF going slightly upward laterally (K2) (Fig. 2).
(G) The location of the upper border of the areola opening is determined through a combination of established guidelines (Fig. 3) as follows:
  - Projection of the IMF to the front of the breast is done by pinching the breast between the thumb and the index finger (Fig. 3). This gives the approximate location of the upper border of the areola opening.
  - Mark the middle of the upper arm; the upper border of the areola opening should be at about this height.
  - Keep in mind the patient’s height and age. In very tall patients, the distance H (Fig. 2) from the jugulum to the future nipple position is larger than in short patients. In young patients, the distance H is shorter than in older patients.
  - If someone with severely ptotic breasts has a long superior medial pedicle and thus is at a higher risk for areola perfusion impairment, it is better to keep the future areola position slightly lower in order to reduce this risk.
(H) H is the distance from the sternal notch to the future upper border of the areola opening (G) (Fig. 2).
(I) Draw a 90° triangle, with the highest point being the future upper border of the areola opening (G). The angle between the vertical sides of the triangle should measure between 80 and 70° (Fig. 4).
  - Draw the 90° triangle with a broken line as a reference guideline.
  - Then mark the sides of an 80° angle with a solid line, giving cranial 4 cm for the areola opening and caudal 6 cm for each of the vertical sides.
  - Length of the vertical lines:
    (i) If the patient is very small, you can also draw the caudal portion as 5 cm.
    (ii) If the patient is at risk for perfusion impairment of the areola due to a long superior medial pedicle, make the vertical lines longer, namely 7–8 cm, in order to make the pedicle wider and optimize blood perfusion.
    (iii) If you perform a one-sided reduction/mastopexy to achieve symmetry, measure the vertical line on the contralateral breast and mark roughly the same length on the reduction side.
  - How to determine the angle between the vertical sides of the triangle:
    (i) In breasts that are not too firm, 80° usually works well.
    (ii) In patients who present with very firm breast tissue, we recommend marking the breast primarily at 70° in order to avoid problems with wound closure due to tension. It can always be enlarged intraoperatively, if necessary, by medial deepithelialization and lateral resection.
    (iii) Always perform the pinch test on the vertical lines. Measure the horizontal medial and lateral lines: J1/J2 and K1/K2 should have approximately the same length. A difference of about 1–2 cm between J1/J2 and K1/K2 can usually be tolerated, while in very large breasts the difference, especially lateral, can sometimes be even larger (Fig. 2).
(J) Horizontal lines medial
(K) Horizontal lines lateral
(L) The cranial 4 cm of the vertical sides of the triangle correlate with the areola opening (Fig. 4). The circumference of the areola opening should match the circumference of the 4 cm areola. You can easily mark the areola opening using a semicircular template. You can create a model using paper or a more rigid material like wood or cardboard with a 4 cm diameter (M).
(M) 4 cm diameter semicircular template (Fig. 4)
(N) Along the upper border of the areola opening there is a dip (G) created where the two semicircles come together (Fig. 4). The two semicircles have to be joined with a straight line (N), which is usually about 0.5 cm higher than the point where the two semicircles originally met. You have to remember this when initially planning the upper border of the areola opening, because the upper border will ultimately be 0.5 cm higher than in your original drawing.

## Summary of Important Points to be Considered in Preoperative Marking

1. If a patient has a firm, parenchymatous breast, use a 70° angle instead of an 80° angle and always check skin tension with the pinch test.
2. If a patient is at high risk for nipple necrosis due to severe ptosis and a long pedicle to the future areola position, set the future areola position somewhat lower and the vertical sides of the triangle longer to make the pedicle wider (up to 7 cm instead of 6 cm, or in high-risk patients 8 cm) in order to ensure pedicle perfusion. If the pedicle is long, it is also advisable to use an angle smaller than 80° for the vertical sides, in order to prevent adverse pressure on the pedicle after wound closure.
3. If you feel a superior medial pedicle is not possible due to severe ptosis and a long pedicle, after preoperative marking and taking the above-mentioned considerations into account you will often see that the pedicle length for superior medial is shorter than it would be for the inferior pedicle. In our opinion, in severely ptotic patients, only after preoperative marking is it possible to accurately estimate whether a superior medial pedicle is a possible or better technique than the inferior pedicle.

## Important Anatomical Landmarks

The superior pedicle is supplied by the descending artery from the internal mammary system, coming usually from the second interspace. It lies in the subcutaneous tissue over the breast parenchyma. The medial pedicle is supplied by a branch that curves around the medial aspect of the breast, usually from the third interspace [8].

The main nerve supply to the nipple and breast skin is the anterolateral branch of the fourth intercostal nerve, which sends a deep branch over the pectoralis fascia and a superficial branch up into the subcutaneous tissue [9, 10]. The deep branch curves up toward the nipple at the breast meridian, and this can be preserved with full-thickness medial and inferior pedicles [8].

## Operative Technique

**Fig. 5:**
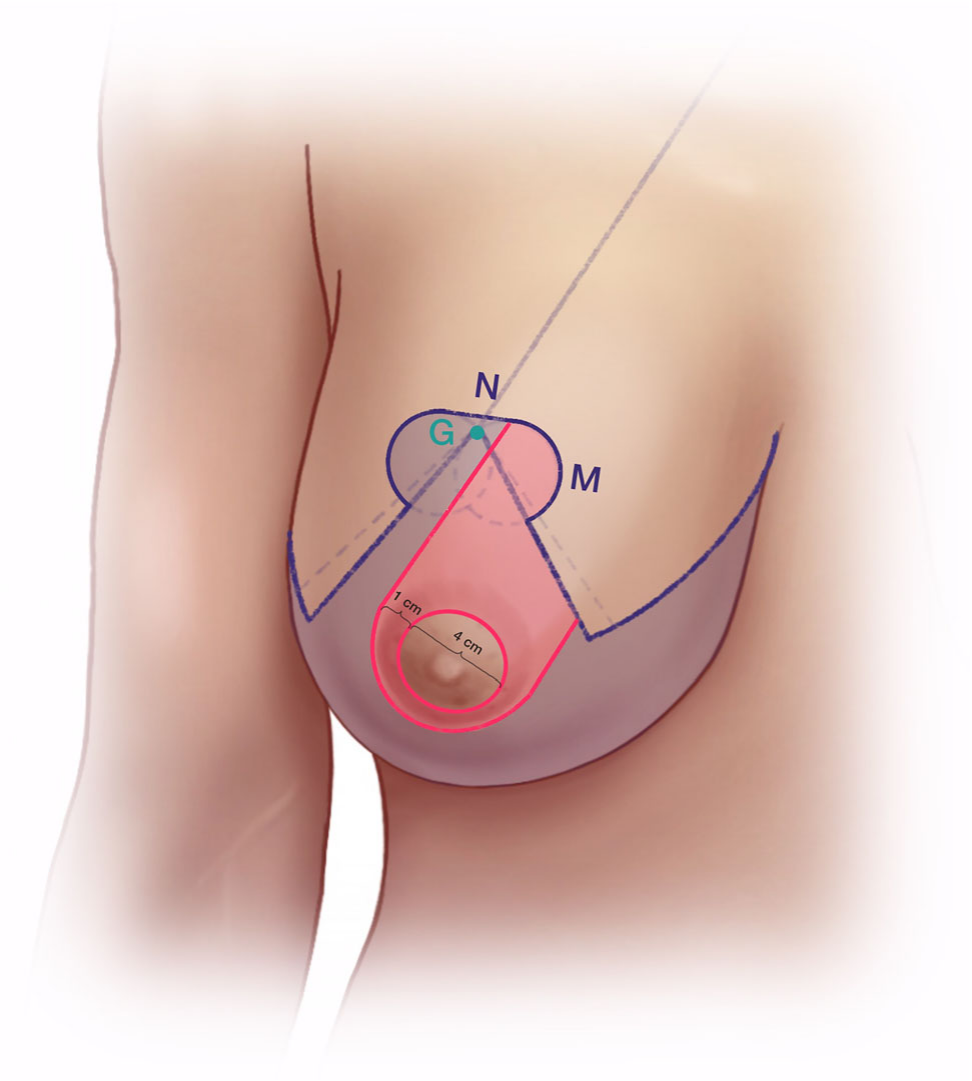
Marking of the superior medial pedicle

**Fig. 6:**
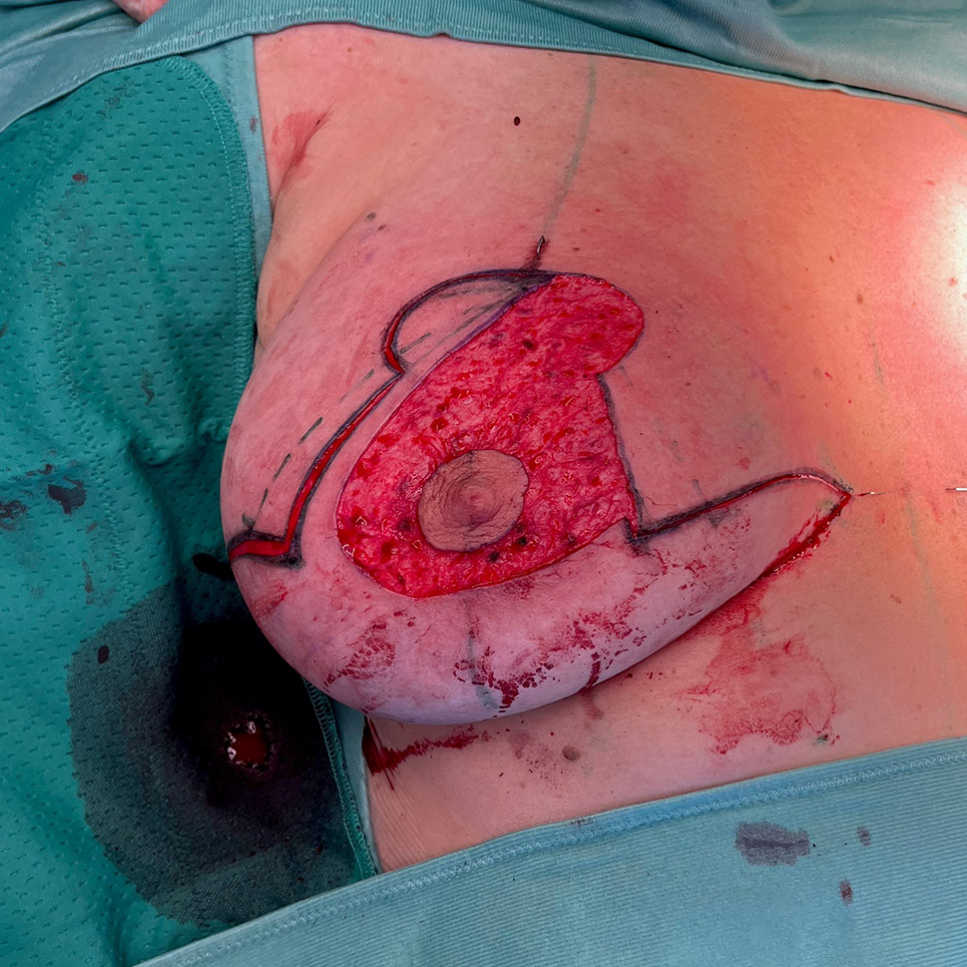
Deepithelialization of the superior medial pedicle

**Fig. 7:**
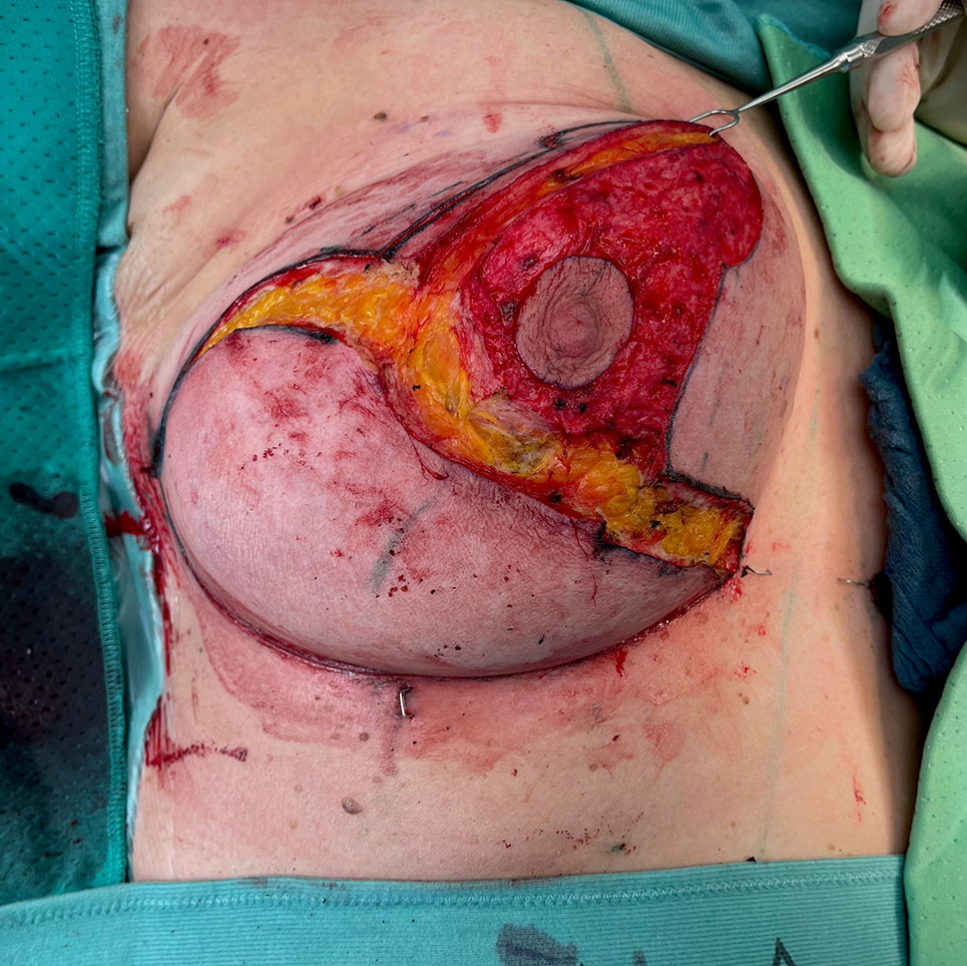
Incision of the whole upper horizontal line

**Fig. 8:**
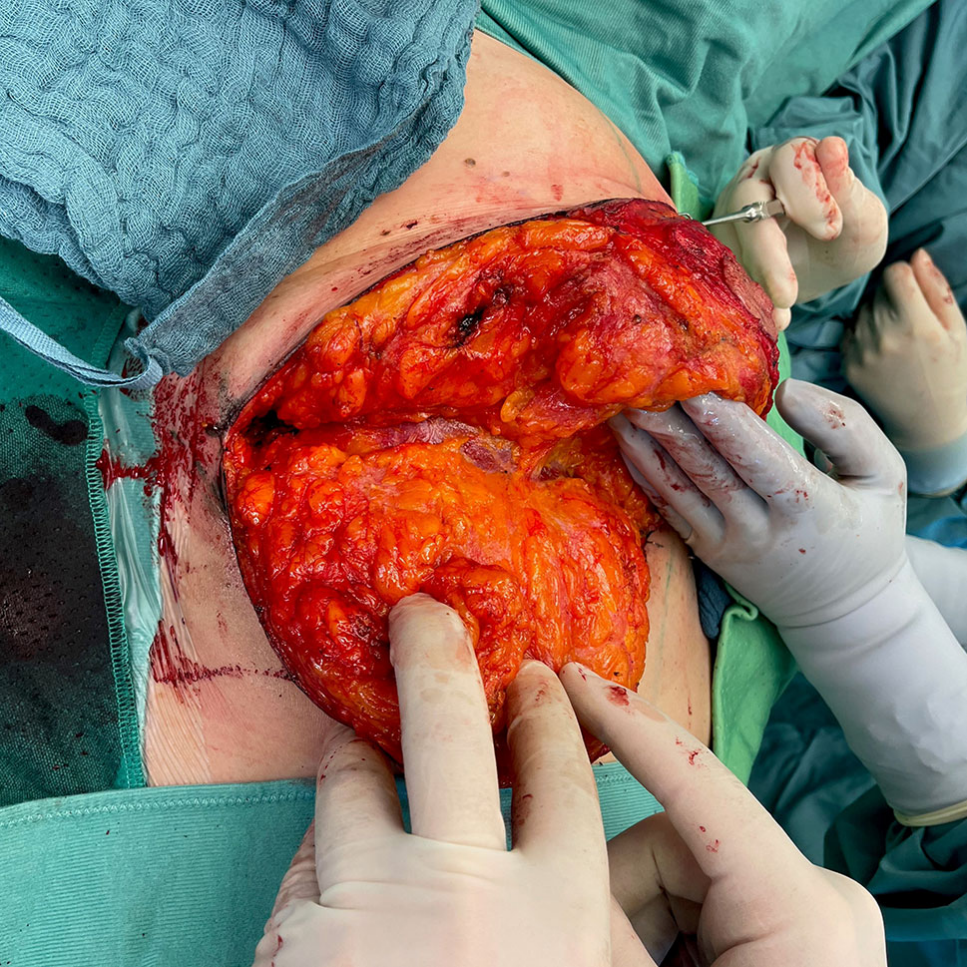
Dissection down to the fascia in the upper horizontal line

**Fig. 9:**
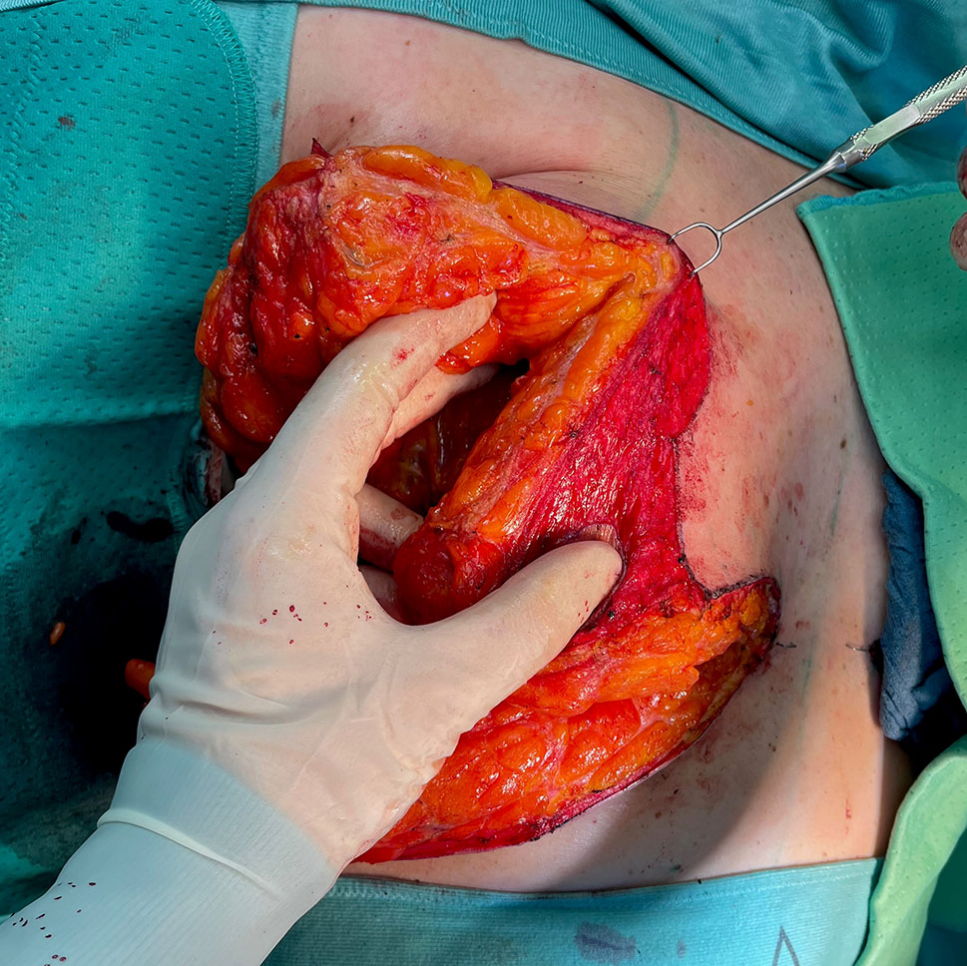
Incision of the lateral part of the pedicle

**Fig. 10:**
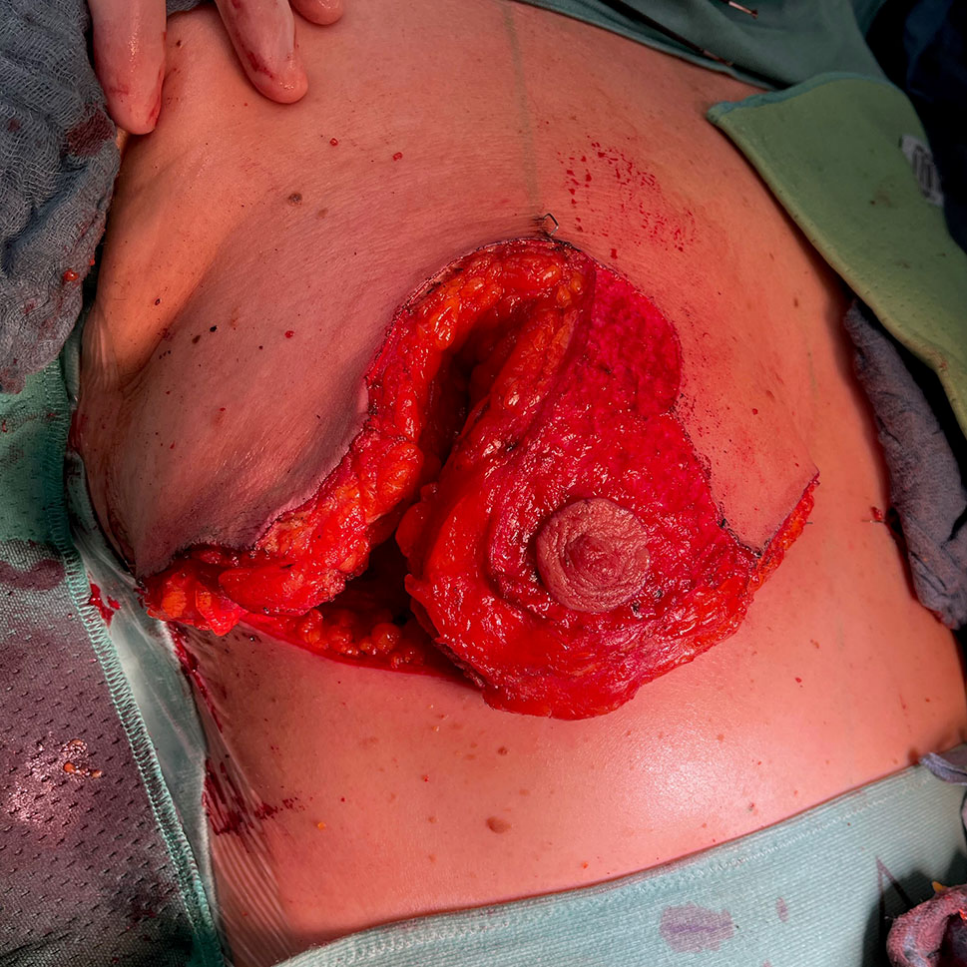
Superior medial pedicle

**Fig. 11:**
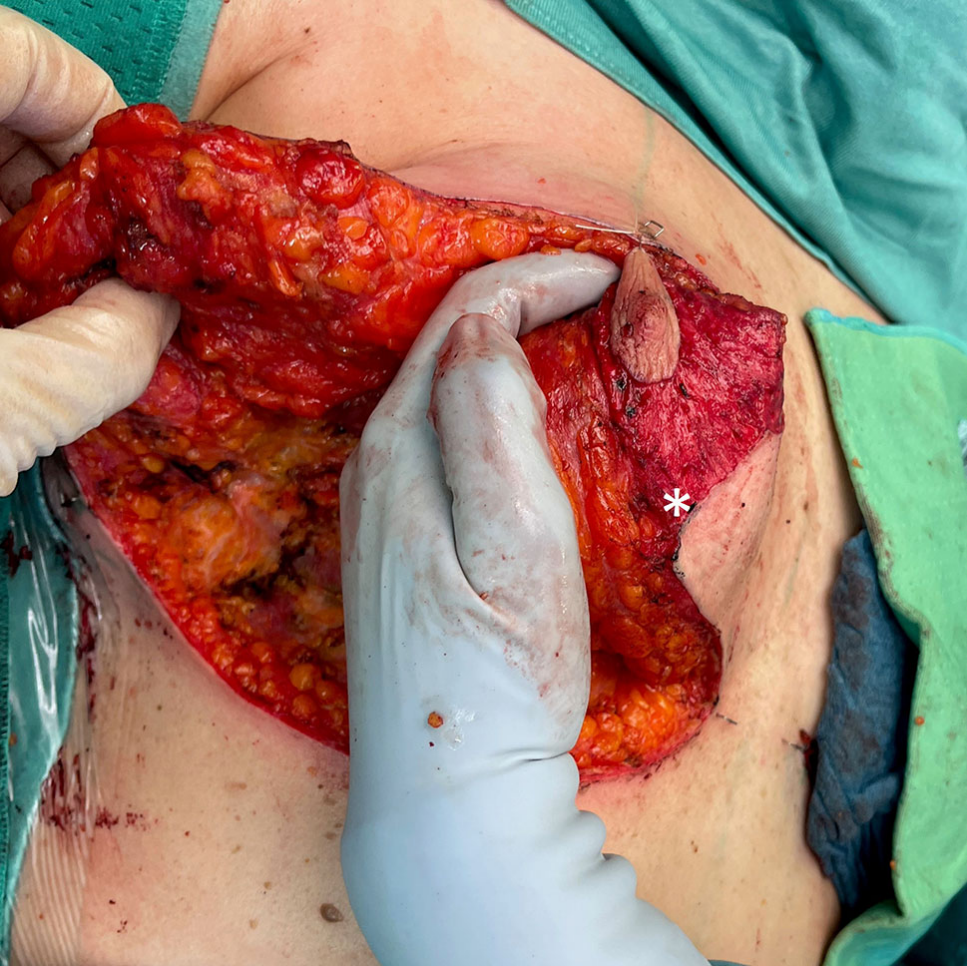
Rotation of the pedicle into its position

**Fig. 12:**
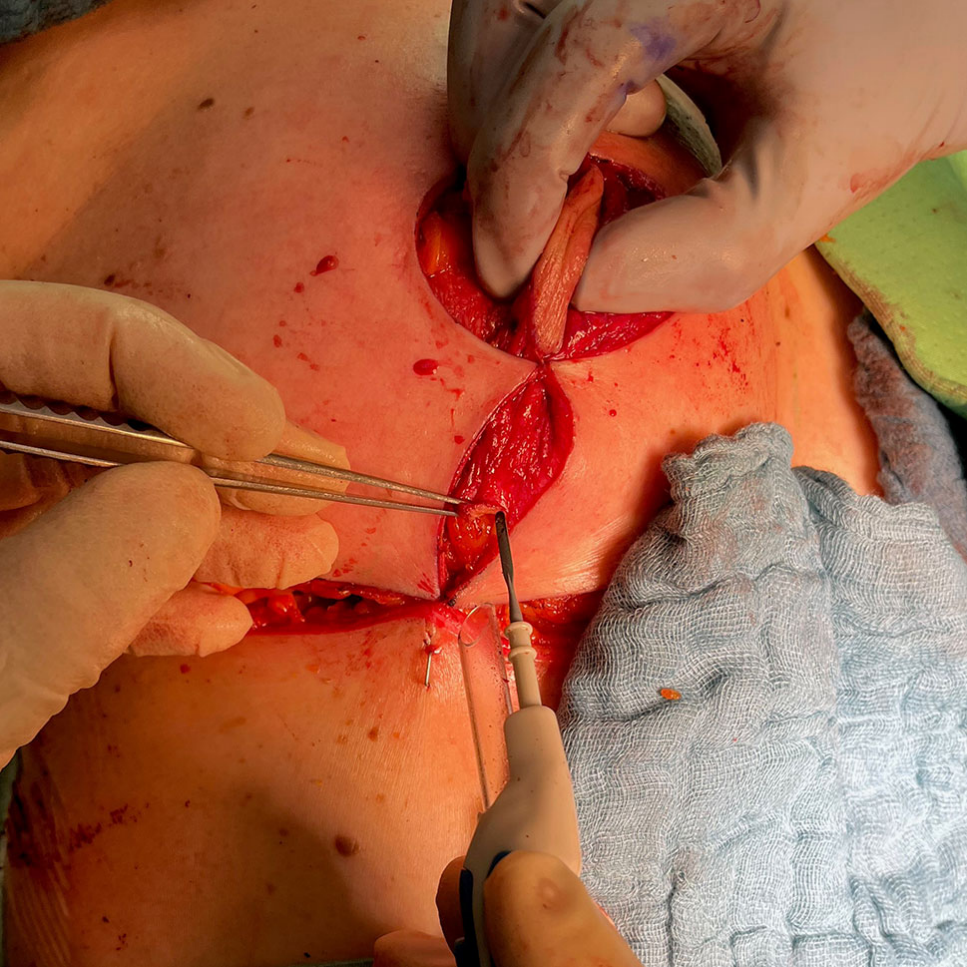
Incision of the dermis at the pedicle inferior medial

**Fig. 13:**
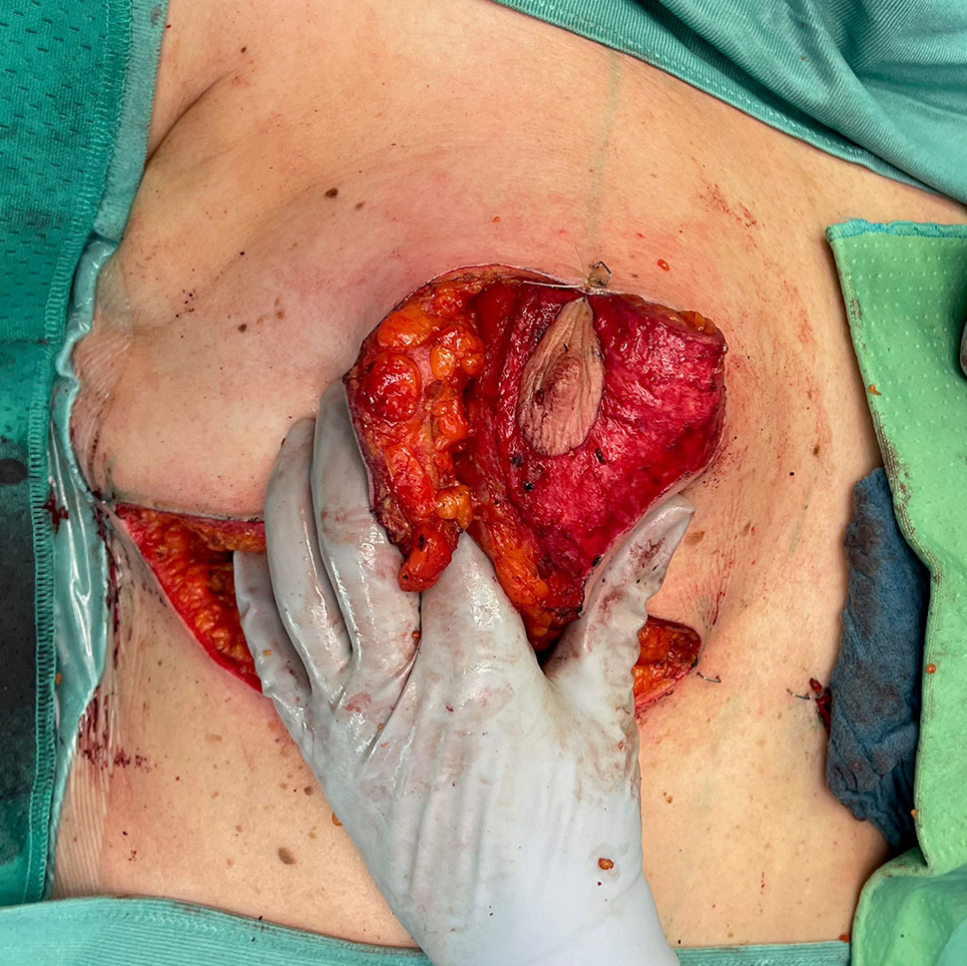
Temporary suture of the superior part of the areola opening

**Fig. 14:**
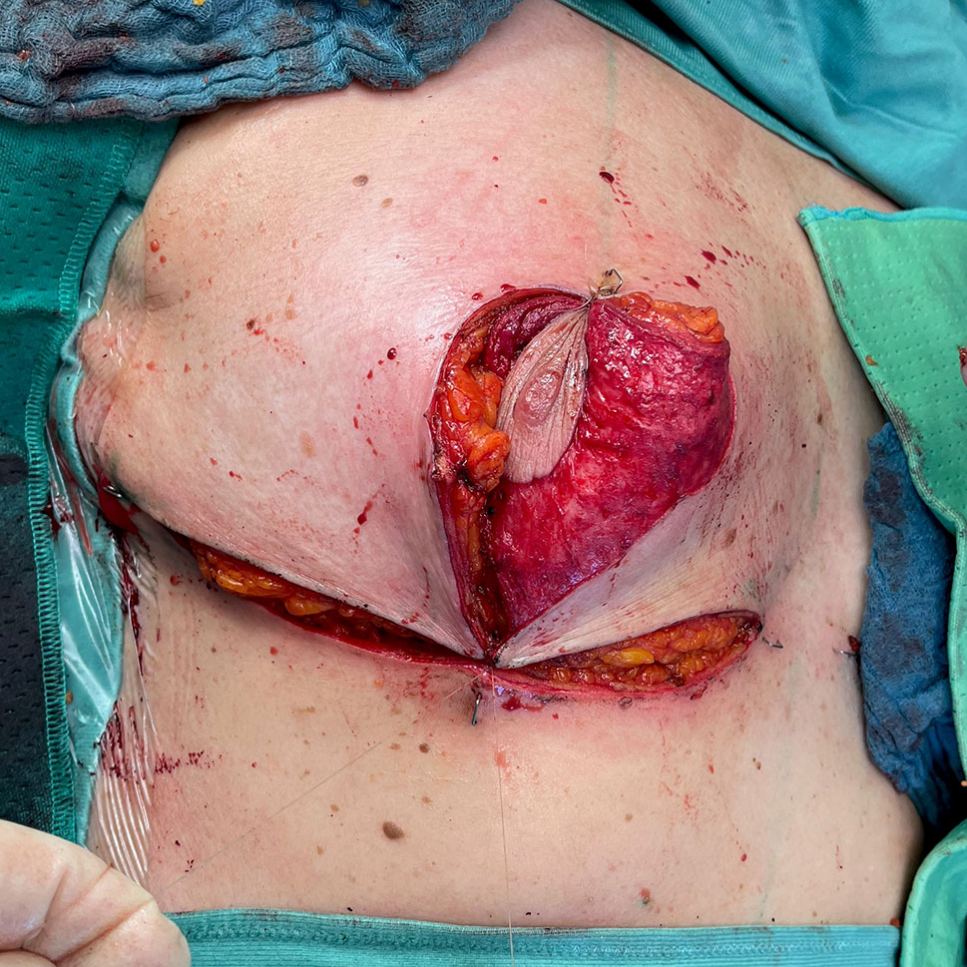
Suture of the inferior triangle region

**Fig. 15:**
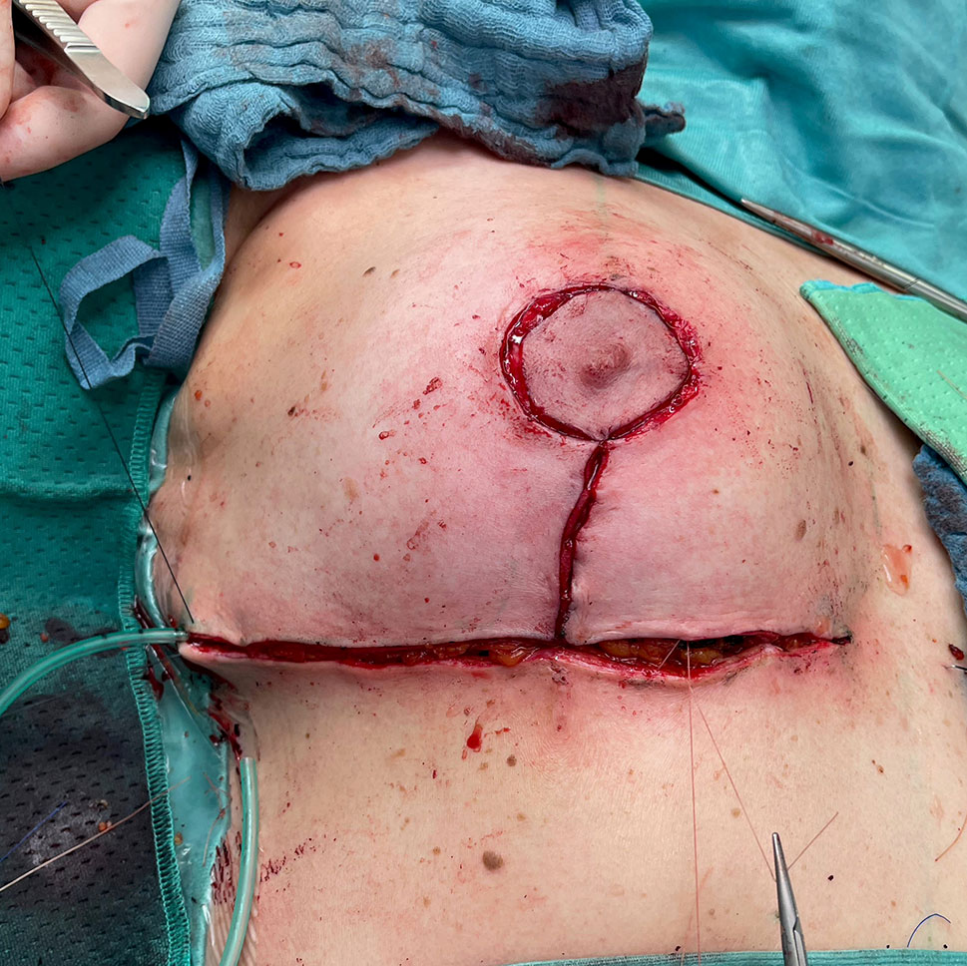
Suture of the medial and lateral parts of the IMF

- Before starting the operation, mark the most medial point (D), the most lateral point (F), Point E on the IMF and the upper border of the areola (G) with skin staples, so that these cardinal points are visible at the end of the operation when you are performing wound closure (Fig. 2). In this way, you can avoid dog ears, especially at the most medial point of the horizontal lines.
- Next, mark the areola with a 4 cm template.
- Mark the superior medial pedicle to include almost the whole possible width, leaving out 0.5 cm at the superior and the inferior end, so that rotation is possible. In the inferior-lateral part of the pedicle include about 1 cm tissue around the areola (Fig. 5).
- Compare the width and the length of the pedicle on both breasts. In order for both breasts to be symmetrical, both pedicles must be roughly equal in width and length.
- Exert tension on both breasts by tightly encircling each breast with a surgical towel and clamping the towel.
- Next, check your areola marking again with the 4 cm template and correct the initial marking, if necessary. When the breast is under tension, like with the surgical towel, the drawing of the areola with the template will be rounder and more exact.
- Incise the areola and the superior medial pedicle with the scalpel and deepithelialize the whole superior medial pedicle (Fig. 6). Then remove the surgical towel.
- Incise all markings on both breasts before starting to work on one side. This will ensure that the markings are still clearly visible on the second breast after performing the breast reduction on the first side.
- Next, start breast reduction on the first side.
- Incise the dermis of the superior medial pedicle on the lateral and inferior parts and leave the medial part intact for the blood perfusion superior medial.
- Then incise the whole upper horizontal line (Fig. 7) and dissect down to the fascia (Fig. 8). It is easier to incise the whole upper horizontal part first before you incise the lateral part of the pedicle, because this gives you better access with more overview of the pedicle.
- Next, incise the lateral part of the pedicle and dissect straight down to the pectoralis fascia (Fig. 9). Always maintain the pedicle in its superior medial position during this manoeuver.
- Remove the lateral tissue according to the preoperative markings. While doing this, keep the areola opening as it will be in its future position in order to not thin out tissue there or leave too much behind. On the one hand, this lateral superior tissue is also part of the breast projection and if you remove too much, you will lose projection. On the other hand, if you remove too little, this tissue can also compress the pedicle and impair blood flow.
- Create the pocket along the fascia. If you start to dissect the pocket laterally just along the fascia, often blunt dissection with your index finger will be possible in the superior part. The pocket should be created mainly superior and lateral. We leave the medial part intact in order to not destroy blood perfusion there.
- The pedicle is full-thickness with the aim of maintaining sensitivity [8] and perfusion from perforators (Fig. 10). We sometimes remove tissue in the upper lateral part of the pedicle in order to facilitate rotation of the pedicle, but leave at least 1 cm tissue thickness in that part so that the superior blood vessel is not damaged. If you maintain the pedicle in its natural superior medial position and compress it with your hands, you will easily see what part of the tissue can be removed. This manoeuver also helps estimate whether more tissue in the inferior part of the pedicle should be removed.
- In our experience, it is advisable to remove the superior lateral part of the planned resection first and to try to rotate the pedicle to its planned position in the created pocket so that you can estimate the future size of the breast before removing the inferior tissue.
- If you see that the breast size is satisfactory in the upper part with the created pedicle, then you can remove the whole inferior part corresponding to the planned resection markings.
- If the breast is too small with only the superior tissue left behind, you can always leave tissue in the lower part behind in order to have enough volume. If necessary, you can leave tissue on the fascia or, if you need more volume, you can deepithelialize the middle inferior part and suture it to the fascia in order to uplift the ptotic inferior tissue. However, this manoeuver is most likely necessary for reduction mastopexy.
- It is also important to leave tissue from the inframammary fold behind, in order to not destroy this important landmark.
- If necessary, you can also remove more breast tissue from the lateral upper part of the breast. Especially in young patients, there is often firm breast tissue laterally, which must then be removed with the reduction.
- After accurate hemostasis control and rinsing with Lavasorb® (polyhexanide), we rotate the pedicle to its planned position, so that its inferior part forms the medial pillar of the vertical sides of the triangle (Fig. 11).
- About 1 cm of the dermis can be incised inferior medial at the pedicle (position marked with * in Fig. 11) in order to facilitate rotation (Fig. 12).
- The surgeon holds the pedicle in its position and the assistant makes one temporary Vicryl® 3-0 suture at the superior part of the areola opening to hold the pedicle in place in the pocket (Fig. 13).
- Next, a Vicryl® 3-0 suture is made at the inferior triangle region in the middle of the IMF (Fig. 14) and then a Vicryl® 4-0 suture at the inferior border of the areola opening to achieve wound closure.
- The assistant holds the pedicle in place, while the surgeon makes three temporary Prolene® 3-0 sutures at 3, 6 and 9 o’clock to join the areola and the areola opening so that the areola is well centered in the opening.
- Next, the areola is secured with a total of eight Vicryl® sutures, and the three Prolene® sutures at 3, 6 and 9 o’clock and the temporary Vicryl® 3-0 suture at 12 o’clock are removed.
- The vertical incision is also sutured with Vicryl® 3-0 and Monocryl® 3-0. Be careful not to make sutures that impair perfusion in the medial part of the vertical incision, where there is also blood inflow into the superior medial pedicle.
- If you need to create more breast projection and you see that the preoperatively marked angle was too small, you can tighten the breast and improve projection with deepithelialization medially (be careful not to impair blood flow to the pedicle medially) and resection lateral to the vertical sides of the triangle. However, with appropriate preoperative markings, this is very rarely necessary.
- After suturing the medial and lateral parts of the IMF, we usually remove the temporary suture at the triangle along the IMF and make it anew. After suturing the medial and lateral parts of the IMF (Fig. 15), it is easier to exactly adapt the wound margins in the inferior triangle, where there is the greatest risk of tension.
- The most medial and the most lateral sutures at the horizontal line are also very important in order to avoid dog ears. However, with precise preoperative marking and when also using surgical staples to mark these cardinal points before incision, you see precisely where you have to make these two stitches without creating dog ears.
- After making the most lateral stitch along the IMF, we insert a 12 redon drainage laterally.
- Subcutaneous sutures are made with Vicryl® 3-0 and Monocryl® 3-0 and skin sutures with resorbable Monocryl® (polyglecapron 25) or Monoderm® (quill monoderm PGA-PCL).
- For the dressing, we use Lomatuell® and Mepore® and a bandage. The bandage is replaced by a compression bra on the first or second postoperative day. The compression bra must be worn for six to eight weeks.
- Intraoperatively a single shot of antibiotic is given.

## Advantages of the Described Technique

In Tables 1 and 2, we outline the advantages of our described technique with pros and cons and list a detailed comparison with other superior medial pedicle inverted T breast reduction techniques described in the literature.

**Table 1:** Preoperative marking—pros, cons and detail comparison with other superior medial techniques

	PROS	CONS	Literature
Preoperative marking	Every little step described and demonstrated through illustrations and photos, simple to follow	Basics are also described	Often only main steps are demonstrated, background experience presumed
Breast meridian	Description how to locate it easily (E)		• 7cm lateral to sternal notch [5]. • 6–8 cm from the sternal notch [11] • Should be drawn through the ideal nipple position, not necessarily through the preoperative nipple position [8, 12]
Upper border areola opening	Combination of established rules described in the literature + own tips according to patient's height, age and breast ptosis (G)		• Mostly established rules are described without additional advice • Many surgeons recommend placing the new nipple at the level of the IMF [5, 13–16] • The center of the new nipple is 1 cm above the IMF, the superior border of the areola is 2 cm above this marking [12] • Ideal nipple position is slightly below the middle position of the breast mound [8] • 10 cm down from the upper breast border and 10 cm from the chest midline [8]
Most medial end of the IMF scar	Advice on how to shift the most medial end of the IMF scar away from the sternal midline (D)		• To our knowledge, not mentioned in the literature • Description as sometimes difficult to avoid [8] • Try to match the length of the skin flaps to the incision of the IMF [8] • Liposuction [8]
Wise pattern skin resection • Angle between the vertical sides • Length of the vertical lines	• Exact angle description (I) with special tips form firm breast tissue (Id) • Advice for adopting the length of the vertical lines according to pt's height and for pts presenting with higher risk of perfusion impairment (Ic)		• Often a wise pattern with a wire template adjusted for each patient described [5] • Wise pattern for the skin that remains as a brassiere to hold the breast shape [8] • The vertical and lateral limbs are marked according to the amount of skin that needs to be removed [15] • The breast is first polled to the medial side and next to the lateral side and two vertical lines are drawn downward forming the triangle [16] • Length of vertical lines is often described as 6 cm [5, 16], sometimes 7 cm [12] • In gigantomastia longer vertical limbs are recommended with the aim to reduce perfusion problems [17]
Areola opening	• Thorough description of how to draw it (G, N) • With a 4 cm semicircular template (M). easy to create • Preoperative marking of the areola opening means shorter operating time		• Some surgeons draw the areola opening preoperatively and some intraoperatively [8, 13] • The circumference of a 5 cm-diameter areola is 16 cm, and the circumference of a 4.5 cm-diameter is 14 cm (original wise pattern) [8] • By only marking a triangle preoperative the position of the nipple-areolar complex may be decided intraoperatively after resection and tailor tacking [13]
Special tips	•How to deal with very parenchymatous breasts, severe ptosis, long pedicles •How to decide if the superior medial pedicle is possible

**Table 2:** Operative technique—pros, cons and detail comparison with other superior medial techniques

	PROS	CONS	Literature
Operative technique	A thoroughly step-by-step description with the aim to easily understand every step	Basics are also described	Often not all steps outlined and demonstrated
Avoid dog ears	Mark the most medial (DJ and most lateral point (F) with skin staples before starting the operation		• To our knowledge this maneuver is not described in the literature • Remove tissue deep to the skin to smooth out dog ears [8]
Mark the superior medial pedicle	Use almost the whole possible width with enact description and presentation how to do it (Fig. 5)		• Some described techniques aim for a very small pedicle[14, 16] • In cases of gigantomastia with longer pedicles a wider flap base is recommended [17]
Tip for achieving symmetry through pedicle size	If both pedicles are roughly equal in width and length		• Logical, but to our knowledge not mentioned in the literature
Check the areola marking again under tension	With this advice the drawing of the areola will be rounder		To our knowledge not described in the literature
Incise all markings on both breasts before starting to work on one side	That way markings are clearly visible on the second breast after operating on the first breast		To our knowledge not explicitly mentioned in the literature
Begin with incision of the whole upper horizontal line down to the fascia before incising the lateral part	Our advice in order to give better access with more overview of the pedicle (Figs. 7,8)		• Mostly the pedicle is created before resecting the parenchyma laterally and inferiorly[8, 13] • Incision of the medial upper horizontal line with limited lateral vertical incision [18] • Beginning with a full-thickness incision on the IMF level reaching the pectoralis fascia[15]
Full-thickness pedicle			Also described in the literature [8]
Remove tissue in the upper lateral part of the pedicle to facilitate rotation of the pedicle	Advice on how to do this easily from our own experience		This removal of tissue also described in films by Hall-Findlay [8] and the literature [11, 17]
Estimate the future breast size before removing the .inferior breast tissue	• Advice to remove the superior lateral part of the planned resection first and rotate the pedicle to its planned position in order to estimate breast size before removing the inferior tissue • This way you avoid resecting too much tissue		To our knowledge not described in the literature
Incision of the dermis inferior medial at the pedicle to facilitate rotation	Our advice with this technique		• To our knowledge rarely mentioned in the literature • Undermining the deep surface of the distal pedicle or scoring the dermis along the inferomedial vertical limb to improve the arc of rotation [13]
Temporary suture at the superior part of the areola opening to hold the pedicle in place	This way the pedicle sets in well in the created pocket (Fig. 13)		• Often small pedicles described and therefore not necessary [14, 16] • The pedicle is fixed to the pectoral is fascia in the superior limit of the upper pole dissection [12]
How to get a rounder areola	By centering the areola well in the areola opening with 3–4 temporary sutures before performing the final 8 sutures		To our knowledge not explicitly described in the literature but useful, especially for residents

## Conclusion

**Fig. 16:**
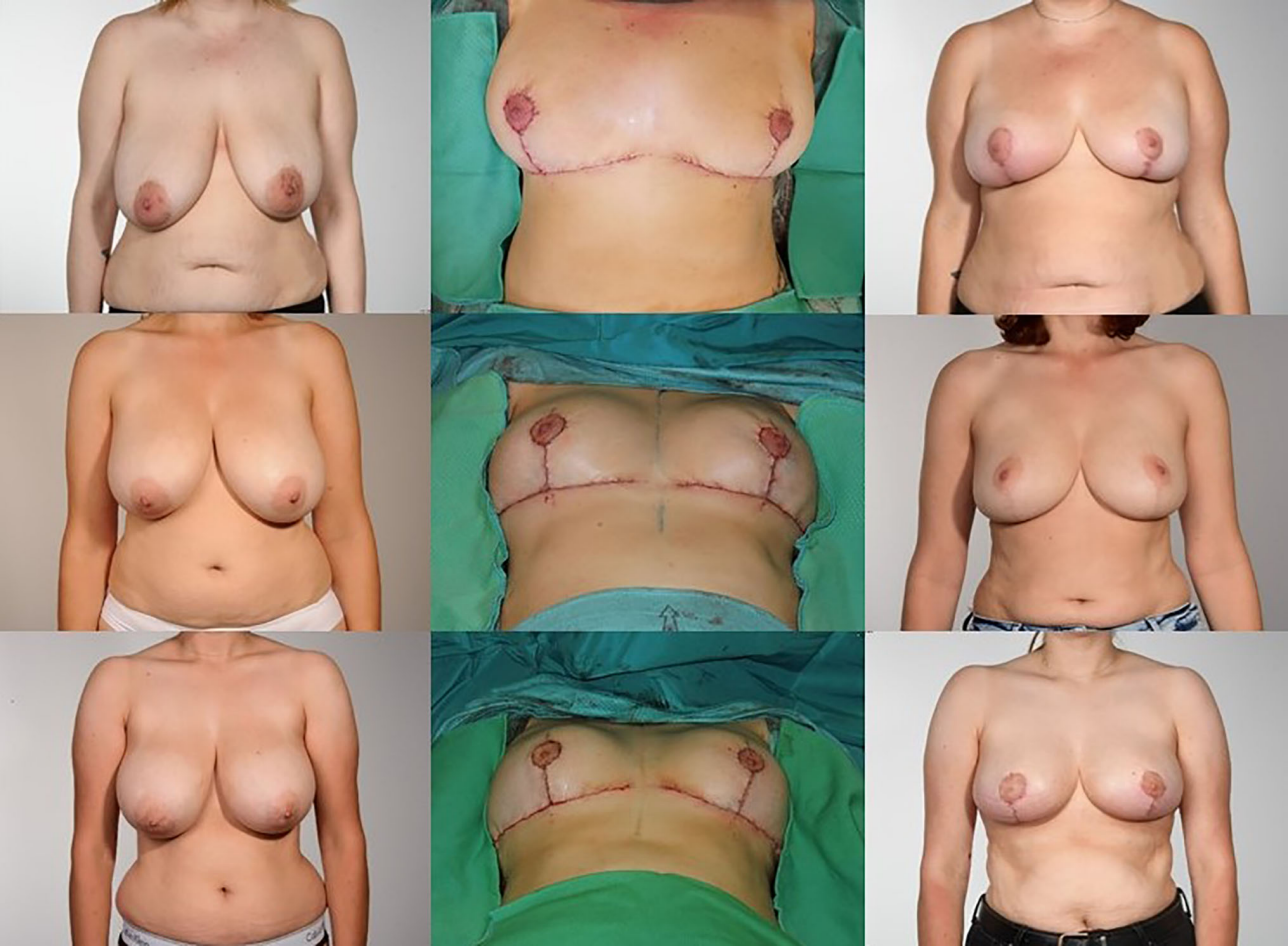
Pre- and postoperative photographs of breast reduction patients operated by us

The described “step-by-step” preoperative marking and operative technique can be used safely and with the outlined important considerations it can be easily adapted for every patient (Fig. 16). With the above-described steps in mind, this operation can also be taught to residents in a simple and straightforward manner.
